# Effects of late gestation heat stress in Buffalo heifers on postnatal growth, thermoregulatory, and suckling behavior responses of their newborn calves under subtropical environment

**DOI:** 10.1007/s11250-025-04514-4

**Published:** 2025-06-16

**Authors:** Amr A. Gabr

**Affiliations:** Department of Animal Production, Faculty of Agriculture, Mansoura University, Mansoura, 35516 Egypt

**Keywords:** Heat stress, Thermoregulatory response, Suckling behavior, Newborn calves, Buffalo heifers

## Abstract

This study aimed to evaluate various growth, physiological, and suckling behavioral parameters of calves born to nulliparous buffalo heifers exposed to summer heat stress (HT, provided only shade, *n* = 12) or winter coldness (CL, *n* = 12) during the final 60 days of gestation. Calves were individually housed and were monitored from birth until 6 days of age. Rectal temperature, pulse rate, and respiratory rate were measured, and heat tolerance and adaptability indices were calculated. Suckling responses, suckling durations, and milk drinking speeds were also recorded. HT calves had lighter birth weights, with approximately 1.26 kg and 1.06 kg differences for male and female calves, respectively. They gained less weight from birth to 6 days, with a difference of 0.12 kg/d for males and 0.11 kg/d for females and weighed about 1.9 kg less at 6 days old. HT calves exhibited elevated rectal temperatures, respiration rates, and pulse rates, with increases of approximately 0.7 °C, 27 breaths/min, and 20.3 beats/min, respectively, compared to CL calves. Female calves were more susceptible to heat stress than males and exhibited lower heat tolerance and adaptability. HT calves exhibited a shorter total suckling duration immediately after birth compared to CL calves by about 55.3% and demonstrated faster milk drinking speeds by about 44%. Only HT female calves exhibited slower milk drinking speeds compared to HT males, with a reduction of about 18.7%. In-utero heat stress during late gestation had negative immediate and prolonged effects on postnatal performance and suckling behavior in buffalo calves, particularly in female calves.

## Introduction

Buffaloes are well-suited for the Egyptian climate, particularly the hot and humid conditions prevalent in many regions. They possess morphological and behavioral characteristics that enable them to efficiently regulate their body temperature in such environments (Napolitano et al. 2023a). Compared to cows, buffaloes demonstrate greater tolerance to heat stress and can better adapt to tropical climates, resulting in fewer adverse effects on their productivity and physiology (Petrocchi Jasinski et al. 2023). This adaptability makes buffalo farming a viable option for farmers in areas where other livestock species may struggle with climate-related challenges.

Heat stress poses a significant challenge to the dairy production industry (Bakony and Jurkovich 2020), as previous studies have demonstrated its adverse effect on the productive and reproductive efficiency of dairy animals. It is worth noting that the severity of heat stress effects can vary depending on the intensity and duration of the heat stress event, as well as the management practices employed to mitigate its impact. Under heat stress conditions, buffaloes undergo various physiological changes, including alterations in rectal temperature, respiratory rate, heart rate, and skin temperature (Mishra 2021). Additionally, changes in behavior serve as an early indicator of thermal discomfort (Bakony and Jurkovich 2020). While numerous studies have defined temperature humidity index (THI) thresholds for heat stress in cattle, there is limited research on the optimal THI values for buffaloes (Petrocchi Jasinski et al. 2023). In buffalo, THI values below 72 are considered optimal, THI between 72 and 79 indicates mild stress, 80–89 signifies moderate stress, and THI values ≥ 90 indicate severe stress (Choudhary and Sirohi 2019).

Predicting animals’ responses to heat stress enables the development of effective management strategies to alleviate heat load. However, various indices have been developed to monitor the heat stress status in different livestock species (Rocha et al. 2019). While THI serves as a valuable instrument for assessing the potential heat stress on animals, their own physiological responses to heat offer a better indication of their level of heat stress (Toledo et al. 2020). Therefore, other indices, such as Benezra’s coefficient of adaptability or the Iberia heat tolerance test, consider vital signs like rectal temperature and respiratory rate in animals.

The late gestation period, particularly the final 60 days before calving, is crucial for nulliparous heifers. This period plays a vital role in ensuring the health and productivity of both the heifer and its offspring. Proper management, nutrition, and attention to the heifer’s needs during this phase are essential for optimal fetal development, mammary gland development, colostrum production, body condition, and long-term productivity and reproduction. Therefore, heat stress exposure during late gestation affects not only the pregnant dam but also the developing fetus, as it coincides with the late gestation stage. Previous studies extensively document the adverse effects of late gestation heat stress on the calf, including compromised fetal growth and development, shortened pregnancy period (Monteiro et al. 2014; Ahmed et al. 2021; Cartwright et al. 2023), and reduced birth weight (Dado-Senn et al. 2020; Ahmed et al. 2021; Cartwright et al. 2023). In utero heat stress also influences postnatal physiology, such as immune function and metabolic adaptation, in the offspring (Monteiro et al. 2016; Dahl et al. 2016; Ahmed et al. 2021).

The notion that heat stress has a milder impact on the nulliparous heifers and their calves’ growth compared to mature dry cows has led to a limited consideration towards understanding the effects of heat stress on nulliparous pregnant dairy heifers and their offspring (Davidson et al. 2021). Currently, it remains unknown whether the intrauterine environment of actively cooled and heat-stressed nulliparous pregnant buffalo heifers exerts similar or different outcomes on the postnatal life of their offspring. Moreover, there is limited information available on the effect of heat stress in buffaloes raised under intensive conditions (Petrocchi Jasinski et al. 2023). Additionally, the suckling behavioral patterns of newborn calves during the early neonatal period have not been adequately studied in response to the effects of heat stress during late gestation. Therefore, this study aimed to investigate the impact of late gestation environmental heat stress in nulliparous buffalo heifers on the growth performance as well as various physiological and suckling behavioral parameters of their calves.

## Materials and methods

The research was conducted at a private herd of dairy buffaloes located in Cairo-Ismailia desert road, Ismailia Governorate, Egypt. The herd was closely monitored throughout the study for research purposes. The research did not include any actions that changed how the farm functioned or impacted the behavior of the buffalo heifers and calves.

### Animals and management

Twenty-four buffalo heifers (*n* = 12 in winter and *n* = 12 in summer, 576 ± 0.53 kg body weight, and 7 months pregnant) were randomly selected from a herd of about 975 buffaloes. All heifers used in the study underwent an estrus synchronization protocol using the prostaglandin F2α analogue (PGF Veyx® forte 50 ml) (cloprostenol sodium 0.250 mg/ml) from Veyx Pharma GmbH–Germany. This was administered intramuscularly at a dose of 0.5 mg cloprostenol/heifer, equivalent to 2 ml of PGF Veyx/heifer, on two occasions with an 11-day interval between treatments. Estrus was continuously monitored following the second PGF2α injection, and heifers were artificially inseminated 12 h after the observed onset of estrus signs. Rectal palpation was performed to assess the heifers’ estrous status at the time of insemination. Pregnancy was confirmed about 40–45 days post-insemination using transrectal ultrasonography (Sonoscape L741 V Linear Rectal Probe Veterinary Transducer 7.5 MHz, China). At the seventh month of gestation, the study heifers were identified after ultrasound re-confirmation of pregnancy status, and the expected calving date was determined. The heifer’s enrollment into the study was based on their body condition and body weight across all heifers that were subsequently designated to calve in either the summer or winter seasons. This ensured that the heifers entering the study had comparable baseline characteristics before they experienced the differing seasonal conditions based on their pre-determined calving times. The study heifers were managed identically together in a single group until parturition in both seasons, housed in semi-open shaded pens with sand bedding, without any water soakers or fans. They were environmentally exposed, for approximately 60 days before the expected calving date, to an average temperature-humidity index (THI) of 55.5 and 81.4 ± 0.46 during the study winter months (December and January) and summer months (July and August), respectively. To assess the environmental conditions, given the physiological similarities between cattle and buffaloes as large ruminants, and in the absence of a more appropriate, validated index for buffaloes, the Dikmen and Hansen (2009) recommended THI equation was used for each season group: THI = (1.8 × T + 32)-[(0.55–0.0055 × RH) × (1.8 × T-26)], where T represents the ambient temperature in °C and RH represents the relative humidity as a percentage. However, during the winter period, the temperatures at the farm varied between 10.4 and 17.5 °C, while in the summer period, they ranged from 25.5 to 36.7 °C (TFA Dostmann GmbH & Co. KG,Wertheim, Germany) using a thermo-hygrometer.

All the buffaloes were given a total mixed ration (TMR) consisting of the same type of diet tailored to their maintenance and production needs. The TMR was mixed daily and adjusted to fulfill the expected requirements of energy, protein, minerals, and vitamins for their reproductive stage and milk production. The TMR underwent monthly analysis. The main analysis revealed that it contained 12.8% crude protein, net energy for lactation (2.6 Mcal/kg), 31.8% neutral detergent fiber, and 18.8% acid detergent fiber. From December to May, the buffaloes were provided with Egyptian clover berseem (*Trifolium alexandrinum*), while Alfalfa hays were given for the rest of the year.

After the heifers gave birth, their resultant calves were immediately separated and weighed. Twenty-four of heifer calves (*n* = 12 in winter and *n* = 12 in summer, with an equal number of males and females) were followed from birth until 6 days of age. These calves were raised in individual pens inside an open-sided barn with sand bedding under identical management conditions. Within the first 2 h of birth, the calves were fed 2 L of high-quality colostrum (i.e., ≥ 22% Brix refractometer reading) came from their respective dams. The daily calves’ feedings consisted of 2 L of milk given twice a day at 0700 and 1800 h throughout the study period. For the first 3 days, the calves continued to be fed colostrum through a nipple bottle. Afterward, during the second 3 days, they were transitioned to standard milk feeding using a milk bucket.

### Physiological parameters

The physiological parameters were assessed in each calf during the first 6 days after birth, both in winter and summer seasons, at 0700 and 1700 h. A digital thermometer (model FT09, Beurer, Germany) with an accuracy of ± 0.1 was used to measure rectal temperature. The observer performed measurements on summer-born calves was blind to the specific values recorded for winter-born calves. The pulse rate, measured in beats per minute, was obtained from the coccygeal artery. The respiratory rate was recorded by visually observing the flank movement of the calves for 1 min, from a distance without causing any disturbance. The recording of the respiratory rate was done first, followed by the pulse rate and rectal temperature, to ensure accurate observations.

### Heat tolerance and adaptability

The heat tolerance index (HTI) was calculated by Iberia heat tolerance test (Rhoad 1944) using the formula: HTI = 100-[18 × (RT—38.3)], where RT is the rectal temperature in °C. If the calculated value of the test is more nearer to 100 then the particular animal is more heat tolerant than others. The adaptability of each animal was evaluated by Benezra’s index of adaptability (BIA) using the formula: BIA = RT/38.3 + RR/23, where RT represents the rectal temperature in °C and RR is the respiration rate per minute (Benezra 1954). A sum of 2 indicates maximal adaptability, while a sum greater than 2 suggests reduced adaptability.

### Behavioral parameters

During the first 6 days after birth, all suckling sessions were closely observed and continuously recorded through direct sight and video recordings. No suckling sessions were missed during the observation period. All behavioral observations were conducted by a single, trained expert observer. The behavioral parameters used in this study were determined based on observed calf suckling behavior. The measurements of nipple bottle suckling behavior parameters were conducted during the initial three days after birth, while the bucket suckling behavior parameters were assessed during the subsequent three days. The following parameters were considered within a suckling session: 1) Suckling response: this refers to the latency it takes for the calf to locate and latch onto the nipple or bucket after it is presented, 2) Actual suckling duration: it represents the total time the calf spends actively suckling the milk (without including any pauses taken during the process), 3) Pause duration: this denotes the time interval during which there is no contact between the calf’s mouth and the nipple bottle or bucket (it also includes instances where the calf engages in activities unrelated to suckling), 4) Total suckling duration: this measures the overall time taken from the beginning of the suckling session until the milk is completely consumed (this duration may encompass one or several suckling bouts), 5) Number of bouts per suckling session: it refers to the count of distinct suckling episodes within a single suckling session. Furthermore, milk drinking speed, calculated by dividing the amount of milk consumed by the duration of its actual suckling period, was measured in liter per minute.

### Growth performance

The birth weights of the calves were recorded, and their weights before the morning suckling were determined at 3 and 6 days of age and the growth rates were calculated. To evaluate the efficiency of milk utilization, milk conversion rates were calculated by dividing the total amount of consumed milk by the total weight gain in kilograms.

### Statistical analysis

All statistical analyses were conducted using the general linear model (GLM) procedure in SAS version 9.3 (SAS Institute Inc., Cary, NC, USA). The model used was as follows: Yijk = μ + Hi + Sj + (HxS)ij + Eijk. In this model, Yijk represents the studied growth and behavioral traits, μ represents the overall mean, Hi represents the effect of heat stress, Sj represents the effect of calve gender, (HxS)ij is the interaction between heat stress and calve gender, and Eijk represents the experimental error. Moreover, another model was employed for studying physiological parameters, which was as follows: Yijke = μ + Hi + Tj + Sk + (HxT)ij + (HxS)ik + Eijke, where Yijk represents the studied physiological traits, μ represents the overall mean, Hi represents the effect of heat stress, Tj represents the effect of time of the day, Sj represents the effect of calve gender, (HxT)ij is the interaction between heat stress and time of the day, (HxS)ij is the interaction between heat stress and calve gender, and Eijk represents the experimental error. The data was presented as least square means along with the standard error of the mean. Calf served as the experimental unit for all the data. Differences were determined based on Tukey-adjusted pairwise comparisons at a significance level of 5%.

## Results

### Birth weight and growth performance

Both heat stress and gender have significant effects on the birth body weight and growth efficiency of newborn calves during their first week after birth (Table 1). It is observed that in both seasons, male calves had heavier (*P* < 0.001) birth body weights, body weights at 3 and 6 days, and higher growth rates compared to female calves. Additionally, it is noticeable that the body weights and growth rates of in-utero cooled calves were higher (*P* < 0.001) than those born from in-utero heat stress. Notably, in-utero cooled female calves had similar body weights and growth rates to in-utero heat stressed male calves. Furthermore, in-utero cooled male calves demonstrated the best milk conversion rate (*P* < 0.001), while in-utero heat stressed female calves exhibited the worst milk conversion rate.

**Table 1:** Birth weight, growth performance, and milk conversion from calves born to nulliparous buffalo heifers; that were exposed to either cooling or heat stress environments (winter and summer seasons) during the last 60 d of gestation

	Winter		Summer		SEM	*p*-value		
	Male	Female	Male	Female		Season	Sex	SxS
Birth weight, kg	40.63^a^	39.53^b^	39.37^b^	38.47^c^	0.075	< 0.001	< 0.001	0.197
Body weight at 3 d, kg	41.83^a^	40.48^b^	40.36^b^	39.12^c^	0.073	< 0.001	< 0.001	0.503
Body weight at 6 d, kg	43.63^a^	41.78^b^	41.65^b^	40.00^c^	0.062	< 0.001	< 0.001	0.075
Daily weight gain 0–3 d, g	0.400^a^	0.317^b^	0.333^b^	0.217^c^	0.010	< 0.001	< 0.001	0.113
Daily weight gain 3–6 d, g	0.600^a^	0.422^b^	0.428^b^	0.294^c^	0.013	< 0.001	< 0.001	0.110
Daily weight gain 0–6 d, g	0.500^a^	0.370^b^	0.381^b^	0.256^c^	0.008	< 0.001	< 0.001	0.738
Total body weight gain, kg	3.000^a^	2.217^b^	2.283^b^	1.533^c^	0.046	< 0.001	< 0.001	0.719
Milk conversion, L/kg gain	7.008^c^	9.492^b^	9.218^b^	13.75^a^	0.233	< 0.001	< 0.001	< 0.001

^*^a, b, and c: means in the same row with different superscripts are significantly different (*p* < 0.05)

### Calves’ physiological response

**Fig. 1:**
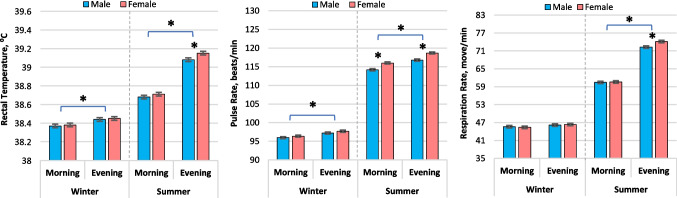
Rectal temperature, respiration rate, and pulse rate from calves born to nulliparous buffalo heifers that were exposed to either cooling or heat stress environments (winter and summer seasons) during the last 60 d of gestation. Data are LSM ± SE, and the symbol (*) indicates that means differ (*p* < 0.05)

Figure 1 illustrated that rectal temperature increased (*P* ≤ 0.002) in both male and female calves during the evenings compared to the mornings in both seasons. There was no distinction in the rectal temperature between male and female calves in the mornings and evenings, except in the heat stressed females which had higher (*P* = 0.026) rectal temperatures than males in the evenings. In both sexes, the pulse rates of calves were higher (*P* ≤ 0.001) in the evenings than in the mornings during both seasons. Additionally, heat stressed female calves exhibited higher pulse rates (*P* < 0.001) than males in both morning and evening. Respiratory rates increased (*P* < 0.001) in both male and female calves from winter to summer. In in-utero cooled calves, respiratory rates did not significantly differ between morning and evening, but in heat stressed calves, the rates were higher (*P* < 0.001) in the evenings compared to the mornings, while being higher (*P* = 0.002) in female calves than males. Overall, the interaction between time of day, gender, and heat stress was not significant for study physiological parameters (*P* ≥ 0.276).

**Fig. 2:**
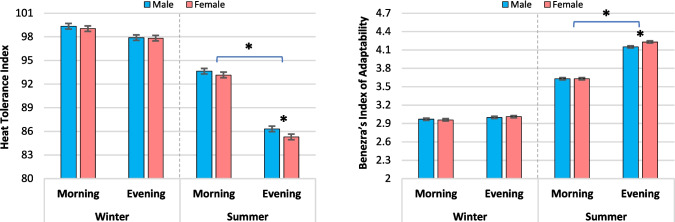
Iberia heat tolerance index and Benezera’s index of adaptability from calves born to nulliparous buffalo heifers that were exposed to either cooling or heat stress environments (winter and summer seasons) during the last 60 d of gestation. Data are LSM ± SE, and the symbol (*) indicates that means differ (*P* < 0.05)

Figure 2 demonstrated that the heat tolerance index was lower in the summer than in the winter (*P* < 0.001). There was a decrease (*P* < 0.001) in the evening values compared to the morning during the summer season, while no significant difference was observed in the winter season. Heat stressed female calves exhibited lower heat tolerance index values (*P* = 0.04) in the evenings compared to males (85.30 vs. 86.32 ± 0.35, respectively). The Benezera’s index of adaptability values were higher (*P* < 0.001) in summer than in winter and increased (*P* < 0.001) between morning and evening in summer, but not in winter. Furthermore, heat stressed female calves had higher values of Benezera’s index of adaptability (*P* = 0.002) than males in the evenings (4.23 vs. 4.15 ± 0.02, respectively). Generally, the interaction between time of day, gender, and heat stress was not significant for heat tolerance and Benezera’s indexes (*P* ≥ 0.283).

### Calves’ suckling behavior response

Table 2 presented significant findings regarding the impact of season on nipple bottle suckling behavior parameters of newborn calves during the first three days after birth. It was observed that all measured suckling durations were shorter in in-utero heat stressed calves compared to in-utero cooled calves (*P* ≤ 0.006). The sex of the calves influenced the suckling response to the nipple bottle, with female calves taking longer time than males (*P* < 0.001) in both in-utero cooled and heat stressed calves by about 34.7% and 46.5%, respectively. The in-utero cooled calves showed higher (*P* < 0.001) number of bouts per suckling than heat stressed calves. Furthermore, the average milk drinking speed increased in in-utero heat stressed calves compared to in-utero cooled calves (*P* < 0.001). Notably, heat stresses female calves showed less average drinking speed of milk (*P* = 0.004) by about 17.9% than males.

**Table 2:** Nipple bottle suckling behavior during the first three days after birth from calves born to nulliparous buffalo heifers; that were exposed to either cooling or heat stress environments (winter and summer seasons) during the last 60 d of gestation

	Winter		Summer		SEM	*p*-value		
	Male	Female	Male	Female		Season	Sex	SxS
Suckling response, min	0.064^bc^	0.098^a^	0.038^c^	0.071^ab^	0.010	0.006	< 0.001	0.986
Actual suckling duration, min	3.081^a^	3.392^a^	2.003^b^	2.377^b^	0.190	< 0.001	0.074	0.869
Paus duration, min	0.495^a^	0.487^a^	0.066^b^	0.074^b^	0.100	< 0.001	0.989	0.938
Total suckling duration, min	3.641^a^	3.977^a^	2.107^b^	2.522^b^	0.264	< 0.001	0.157	0.882
Number of bouts per suckling	4.267^a^	4.467^a^	1.800^b^	1.967^b^	0.268	< 0.001	0.495	0.950
Average milk drinking speed, L/min	0.551^c^	0.500^c^	0.815^a^	0.669^b^	0.033	< 0.001	0.004	0.161

^*^a, b, and c: means in the same row with different superscripts are significantly different (*P* < 0.05)

Table 3 revealed significant impact (*P* < 0.001) of season on bucket suckling behavior parameters of newborn calves during the second three days after birth. The results showed that heat stressed calves had shorter bucket suckling durations compared to in-utero cooled calves (*P* < 0.001). However, in-utero cooled and heat stressed female calves took longer time for suckling response by about 38.5% and 46.8% than males (*P* = 0.014), respectively. The in-utero cooled calves showed higher (*P* < 0.001) number of bouts per suckling than heat stressed calves. Additionally, the average milk drinking speed increased (*P* < 0.001) in in-utero heat stressed calves compared to in-utero cooled calves, while being lower (*P* = 0.040) in heat stressed female calves by about 19.4% than males.

**Table 3:** Bucket suckling behavior during the second three days after birth from calves born to nulliparous buffalo heifers that were exposed to either cooling or heat stress environments (winter and summer seasons) during the last 60 d of gestation

	Winter		Summer		SEM	*p*-value		
	Male	Female	Male	Female		Season	Sex	SxS
Suckling response, min	0.083^b^	0.135^a^	0.025^d^	0.047^c^	0.017	< 0.001	0.014	0.104
Actual suckling duration, min	1.732^a^	1.893^a^	0.930^b^	1.115^b^	0.097	< 0.001	0.078	0.901
Paus duration, min	0.240^a^	0.275^a^	0.000^b^	0.002^b^	0.052	< 0.001	0.725	0.754
Total suckling duration, min	2.056^a^	2.303^a^	0.955^b^	1.164^b^	0.153	< 0.001	0.130	0.836
Number of bouts per suckling	2.867^a^	3.233^a^	1.000^b^	1.067^b^	0.272	< 0.001	0.427	0.582
Average milk drinking speed, L/min	1.249^c^	1.231^c^	2.432^a^	1.960^b^	0.118	< 0.001	0.040	0.057

^*^a-d: means in the same row with different superscripts are significantly different (*P* < 0.05)

**Fig. 3:**
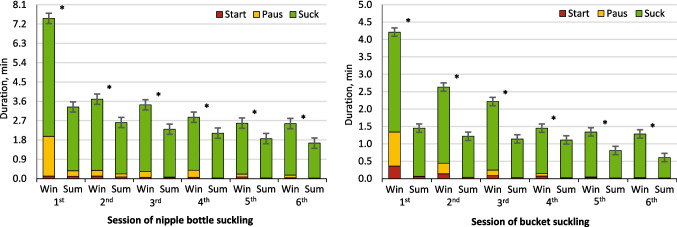
Suckling behavior patterns during the first week after birth from calves born to nulliparous buffalo heifers that were exposed to either cooling or heat stress environments (winter and summer seasons) during the last 60 d of gestation. Data are LSM ± SE, and the symbol (*) indicates that means differ (*P* < 0.05)

Figure 3 illustrated the suckling behavior patterns of newborn calves for each suckling session during their first week after birth. It is evident that, throughout the first three days when using a suckling nipple bottle, in-utero cooled calves required more total suckling duration (*P* < 0.001) compared to heat stressed calves for each suckling session, especially in the initial suckling immediately after birth (7.47 vs. 3.34 ± 0.24 min, respectively). The same pattern persisted when transitioning to suckling from a bucket. The in-utero cooled calves consistently took more total suckling duration (*P* < 0.001) than heat stressed calves in all suckling sessions, and the notable disparity became most apparent during the first suckling from the bucket on the fourth day of calves’ age (4.21 vs. 1.45 ± 0.12 min, respectively).

**Fig. 4:**
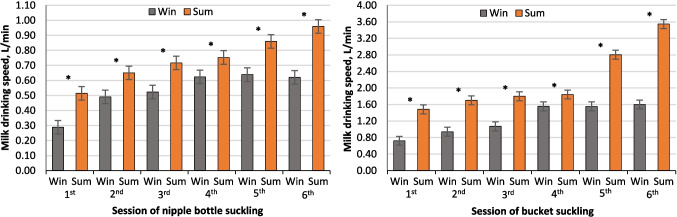
Milk drinking speed during the first week after birth from calves born to nulliparous buffalo heifers that were exposed to either cooling or heat stress environments (winter and summer seasons) during the last 60 d of gestation. Data are LSM ± SE, and the symbol (*) indicates that means differ (*P* < 0.05)

Although the mean milk drinking speed of both seasons’ calves increased linearly from birth to day 6, the in-utero heat stressed calves drank milk at a faster rate (*P* < 0.001) than in-utero cooled calves in all suckling sessions (Fig. 4). In the initial suckling session using a nipple bottle, the in-utero heat stressed calves consumed milk more rapidly (0.514 ± 0.05 L/min; *P* < 0.001) compared to the in-utero cooled calves (0.288 ± 0.05 L/min). During the second suckling session, the drinking speed increased by 0.203 and 0.136 ± 0.02 L/min for the in-utero cooled and heat-stressed calves, respectively. In the final session of nipple bottle suckling, the in-utero cooled calves exhibited a slower (*P* < 0.001) drinking speed (0.620 ± 0.05 L/min) compared to the in-utero heat stressed calves (0.959 ± 0.05 L/min). The same trend persisted when calves had transitioned to suckling milk from a bucket. In the initial session of bucket suckling, the in-utero heat stressed calves consumed milk at a faster rate (1.481 ± 0.108 L/min; *P* < 0.01) compared to the in-utero cooled calves (0.722 ± 0.108 L/min). The milk drinking speed of the in-utero heat stressed calves increased at a higher rate throughout the bucket suckling days, and the drinking speed exhibited a steeper slope on day 6 of the suckling period (*P* < 0.001) compared to the in-utero cooled calves (3.547 vs. 1.600 ± 0.108 L/min, respectively).

## Discussion

While raising buffalo in Egypt offers farmers a sustainable and profitable livestock venture, the prevailing notion of buffalo’s ability to tolerate hot conditions has led to management systems that prioritize efficiency and profitability, often at the expense of animal welfare. These systems often lack proper ventilation, shade, and cooling mechanisms, leaving buffalo vulnerable to heat stress. Buffaloes experience greater discomfort from solar radiation and high temperatures due to their dark coat, sparse sweat glands relative to skin area, and thick epidermal skin layer, which impede their ability to effectively lose heat through evaporative cooling (Hussein et al. 2024). Numerous studies have investigated nutritional interventions and feed formulation adjustments, especially concerning energy and protein, to alleviate heat stress in buffaloes and optimize their production (Paengkoum et al. 2021). Nevertheless, effective heat load control strategies in buffaloes housing remain a critical need for the animals’ well-being. A combined approach of improving both feeding and housing systems will help buffaloes to minimize production losses and enhance reproduction under hot conditions (Omran 2024). Therefore, to ensure the long-term productivity and sustainability of the buffalo dairy industry, it is crucial to implement adequate measures for heat stress management in buffalo, comparable to those applied to other dairy animals, in order to optimize productivity and prioritize animal well-being. In addition, heat stress abatement in calf rearing is often overlooked but it is vital for the overall welfare and prosperity of the dairy herd, represents an essential aspect that requires attention. However, increasing evidence suggests that environmental stress can affect the uterine conditions of pregnant dams and thus can indirectly influence the calf fetus, triggering adaptive responses. These adaptations can persist into the postnatal period and are commonly referred to as "fetal programming" (Bakony and Jurkovich 2020).

The impact of maternal heat stress on fetal development has been extensively investigated, leading to a deeper understanding of the adaptive responses exhibited by the fetus (Bakony and Jurkovich 2020). However, from a practical standpoint, Dado-Senn et al. (2020) recommended to closely monitor calves when THI ranges from 65 to 69 in order to minimize the risks associated with heat stress-related impairments. In the current study, calves experienced during both the in-utero and postnatal periods to environmentally average THI of 55.5 and 81.4 ± 0.46 during the winter months and the summer months, respectively. Therefore, the present study investigated the effects of in-utero heat stress on postnatal various growth, physiological, and suckling behavioral parameters in newborn calves.

### Birth weight and growth performance

As expected, the study results demonstrated that heat stress had an impact on the birth weight of calves. Calves exposed to in-utero heat stress had lighter birth weights than those exposed to in-utero cooling, with differences of approximately 1.26 and 1.06 kg for male and female calves, respectively. This finding aligns with previous research, which has consistently shown that heat-stressed cows in late gestation experience reduced placental function and gestation length, which partly explain lighter calf birth weight (Monteiro et al. 2014; Almoosavi et al. 2020; Dado-Senn et al. 2020; Ahmed et al. 2021; Davidson et al. 2021; Cartwright et al. 2023; Yadav et al. 2024). However, heat stress not only impairs the supply of nutrients from the uterus but also disrupts the heat exchange between the dam and the fetus. Due to the higher metabolic rate of the fetus compared to the dam, even a slight temperature difference can result in fetal hyperthermia (Bakony and Jurkovich 2020). Hyperthermia-induced placental insufficiency compromises fetal growth by causing a reduction in placenta size and function, thereby restricting the exchange of nutrients between the dam and the fetus and modifies genes involved in placental gas and oxygen transport (Bakony and Jurkovich 2020; Casarotto et al. 2025). This can lead to a shortened period of rapid fetal growth and a decrease in birth weight, even with a slight reduction in gestation length, which is commonly observed due to heat stress (Dahl et al. 2016). In fact, Dado-Senn et al. (2020) highlighted that heat-stressed dams tend to calve around two days earlier than their cooled counterparts due to late-gestation heat stress, which likely contributes to the 3% difference in birth weight observed in the current study. Additionally, aside from reduced birth weights, calves exposed to in-utero heat stress also exhibited lower organ masses, including the heart, liver, and kidneys, compared to in-utero cooled calves (Ahmed et al. 2021).

The obtained findings of comparable body weights and growth rates between in-utero cooled female and in-utero heat-stressed male calves present a particularly intriguing finding that necessitates additional investigation. However, several potential mechanisms could contribute to this unexpected likeness. For instance, the THI during the study’s summer months for dams carrying male calves might have impaired placental function and nutrient transfer to a degree that their growth and development trajectory mirrored that of in-utero cooled female calves. Current findings also raise questions about potential sex-specific vulnerabilities to thermal stress during gestation in buffaloes, since limited research exists in this area. Future research with a larger sample size and investigations into the underlying hormonal, metabolic, and potentially epigenetic mechanisms are crucial to fully elucidate the complex interplay between the in-utero thermal environment and offspring sex on growth and development in buffaloes. Moreover, the long-term developmental consequences of these early calves’ growth patterns also warrant further investigation.

Apart from the immediate effects of heat stress experienced at the time of testing, previous research has highlighted the long-term impacts of in-utero heat stress on the production, fertility, and health of offspring, considering the perspective across multiple generations (Halli et al. 2021; Kipp et al. 2021; Yin et al. 2022). It is worth noting that calf birth weight, which is influenced by in-utero heat stress, reflects an early stage of development, occurring directly at birth. This implies that the effects of in-utero heat stress may have a relatively short duration when considered from an across-generation perspective (Yin et al. 2023). However, there is a genetically positive correlation between birth weight and subsequent body weights at later stages, such as weaning weight, insemination weight, and weight at first calving (Yin and König 2018; Easa et al. 2022).

### Physiological response

In the current study, calves experienced similar THI levels during both the prenatal and postnatal periods. The findings of the study provide compelling evidence that calves born to heat-stressed heifers suffer from significant impairments in thermoregulation. The in-utero heat stressed calves exhibited postnatal elevated rectal temperatures, respiration rates, and pulse rates, with increases of approximately 0.7 °C, 27 breaths/min, and 20.3 beats/min, respectively, during the evening compared to the in-utero cooled calves. Moreover, the heat tolerance index consistently exhibited lower values in heat stressed calves, especially during the evenings. These indications of severe heat stress emphasize the detrimental impact on the calves in this study. The present findings align with previous studies on shade heat stress mitigation in hutch-housed dairy calves, demonstrating that postnatal heat stressed calves exhibited heightened thermoregulatory responses (Peña et al. 2016; Kovács et al. 2018; Dado-Senn et al. 2020).

Previous studies investigating adaptive responses of calves in different thermal environments, ranging from neutral/shaded to hot/noncooled conditions, have shown an approximate 50% increase in average respiratory rates as an indication of heightened evaporative cooling efforts (Bakony and Jurkovich 2020). For instance, respiratory rates have been reported to increase from 47 to 53 (de Lima et al. 2013), from 50–78 to 73–105 (Peña et al. 2016), or from 30–50 to 70–140 (Kovács et al. 2018). Moreover, in the face of heat stress, animals instinctively raise their heart rate to effectively transfer heat from their body’s core to the skin surface. Thus, the alteration in heart rate can act as a safeguarding mechanism for animals, enabling them to preserve their heat balance and regulate body temperature efficiently (Wang et al. 2020). Therefore, the elevation in heart rate could depend on the increased blood flow to the skin, which aids in facilitating heat dissipation (Petrocchi Jasinski et al. 2023).

Despite being exposed to identical in-utero THI and being kept in identical conditions after birth, female calves born from heat-stressed buffalo heifers exhibited consistently higher rectal temperature, pulse rate, and respiration rate in contrast to male calves during the first week after birth. Strikingly, these present findings highlight a significant gender difference in response to heat stress, with female calves exhibiting greater susceptibility to its adverse impacts, while males demonstrated superior adaptability. The superior adaptability observed in male calves from heat-stressed dams might be attributed to a more efficient development of cardiovascular control or a lower baseline metabolic rate or the hypothesize that estrogen might have a less pronounced thermoregulatory effect in neonates compared to testosterone. However, variations in sex-dependent characteristics, such as body composition, hormonal regulation, and other sex factors, have been extensively studied for their impact on various aspects of thermoregulation, consequently affecting energy balance (Fernández-Peña et al. 2023). Notably, heat stressed female calves displayed even lower heat tolerance index values compared to males, indicating a heightened vulnerability among females to the detrimental impacts of heat stress. Moreover, the adaptability index, as assessed using Benezera’s index, was higher in heat stressed female calves compared to males, further accentuating the reduced coping ability of heat stressed females and emphasizing their increased susceptibility to the physiological consequences of heat stress experienced during the prenatal period. This gender disparity underscores the lasting physiological implications of in-utero heat stress. Overall, further research focusing on the ontogeny of thermoregulatory mechanisms and the influence of sex hormones in buffalo calves under thermal stress is warranted to fully elucidate these gender-specific responses.

Prior research has demonstrated that in-utero heat stress also exerts carryover effects on postnatal physiology and performance, including immune function and metabolic adaptation, in newborn calves (Monteiro et al. 2016; Dahl et al. 2016; Ahmed et al. 2021). Additionally, Laporta et al. (2017) proposed that the effects of heat stress experienced in-utero can have lasting impacts on the thermoregulatory capacity of calves throughout the preweaning period. In a study by Davidson et al. (2021), slight variations in postnatal thermoregulation measures were observed between in-utero heat stressed and cooled calves born to nulliparous Holstein heifers. On the other hand, based on Napolitano et al. (2023b) findings, the birth weight of water buffaloes influences their thermoregulation in the early days of calves’ life.

### Suckling behavior response

The results of this study indicate that in-utero conditions during late pregnancy of buffalo heifers have a significant impact on the postnatal suckling behavior of newborn calves. The in-utero heat stress calves exhibited a shorter total suckling duration immediately after birth compared to in-utero cooled calves by about 55.3%, and their milk drinking speed was observed to be faster by about 44%. The same pattern extended throughout the experimental period for all suckling sessions. The current heightened suckling response, shorter suckling durations, reduced number of bouts, and accelerated milk drinking speed observed in heat stressed calves can be attributed to the thirst-inducing effect of heat stress on newborn calves. However, the current study’s findings point to heightened physiological responses in heat-stressed calves, with thermoregulation relying on elevated respiratory rates. This mechanism is known to elevate extracellular fluid osmotic pressure, activating the thirst center in the hypothalamus (Kamal et al. 2016) and increase newborn calves’ body water losses (Wickramasinghe et al. 2019). This is potentially exacerbated by the widespread practice among producers, as evidenced in this study, of restricting water access to newborns due to diarrhea concerns (Kertz et al. 2017), which could further compound dehydration and thirst in these newborn calves during high-temperature conditions and explain observed suckling behaviors. Consequently, heat-stressed calves primarily rely on milk to meet their water requirements and compensate for total hydration requirements, causing the observed disparity in their suckling behavior. However, the scarcity of prior research specifically on milk intake rate in calves, especially under heat stress, differs from the focus of most studies on calves’ milk and replacer consumption, which suggests reduced intake due to heat stress around birth (Dado-Senn et al. 2021, 2022). Considering that the current study provided a consistent milk volume across seasons, further investigations into milk consumption rate under varying conditions are necessary.

The influence of calf sex on suckling behavior was also evident in this study, with female calves consistently displaying a longer time to suckling response in both in-utero cooled and heat stressed conditions, with a respective increase of approximately 34.7% and 46.5%, respectively. Notably, only female calves subjected to heat stress exhibited slower milk drinking speeds compared to males, with a reduction of approximately 17.9% and 19.4% during nipple bottle and bucket suckling periods, respectively, suggesting that heat stress may have a more pronounced impact on suckling behaviors in female calves. This gender effect outcome can be attributed to the variances in birth weights, as well as physiological and behavioral differences between male and female calves. Regarding birth weights, calves with lighter birth weights exhibited reduced intake of colostrum compared to calves with heavier birth weights (Mota-Rojas et al. 2022; Napolitano et al. 2023b), and displayed slower milk drinking speed (Montes et al. 2023). Furthermore, the altered suckling response and milk drinking speed observed in heat-stressed female calves are probably associated with and support the current findings indicating that female calves are more susceptible to the adverse effects of heat stress. Moreover, heat-stressed calves altered suckling behavior might be connected with broader physiological stress, including oxidative stress. Numerous studies have indicated that oxidative stress resulting from heat stress has become a well-known determinant of animal performance and health (Purba et al. 2022). Such stress in newborns could cause lethargy or metabolic changes influencing suckling motivation and strength. Although this research did not assess oxidative stress, the high THI exposure likely triggered physiological responses impacting behavior. Future research should directly explore the link between thermal stress, oxidative status, and suckling behavior in buffalo calves.

Generally, it is crucial to consider the current obtained findings, as preweaning calf feeding behaviors have implications for the heifer calves’ long-term performance and the ability to predict their milk yield during their first lactation (Swartz and Petersson-Wolfe 2023). Moreover, considering the influence of environmental and biological factors may enhance the accuracy of predictive models (Cantor et al. 2022). However, in order to gain a more precise understanding of THI effects on calves suckling behavior, further controlled experiments examining the associations between different levels of pre- and postnatal THI exposure and the suckling behavior of newborn calves are necessary.

## Conclusion

According to obtained results, exposure of buffalo heifers to environmental heat stress during late gestation resulted in the birth of lighter calves, and this heat stress had a prolonged impact on the growth rate, physiological response, and milk suckling behavior of the calves. Notably, female calves were more susceptible to heat stress than males and exhibited lower heat tolerance and adaptability, suggesting that male calves have a greater ability to cope with challenging heat stress conditions. While there were no differences in suckling behavior between heat-stressed female calves and males, their milk drinking speed and ability to convert milk was significantly affected. Therefore, it is recommended to invest in management systems aimed at minimizing the impact of heat stress on buffalo dams, particularly during late gestation, as well as on calves during the early weeks of life. Although these investments may incur costs, the long-term benefits are likely to outweigh them.

## Data Availability

The data obtained during the current study are presented in the manuscript and available on reasonable request.
